# Intelligent Bimetallic Nanoagents as Reactive Oxygen Species Initiator System for Effective Combination Phototherapy

**DOI:** 10.3389/fbioe.2020.00423

**Published:** 2020-05-08

**Authors:** Hongfeng Li, Ying Li, Jingjing Xiang, Xing Yang, Chunbing Li, Chuangjun Liu, Qi Zhao, Lihua Zhou, Ping Gong, Jiahao Huang

**Affiliations:** ^1^Biomaterials Research Center, School of Biomedical Engineering, Southern Medical University, Guangzhou, China; ^2^Guangdong Key Laboratory of Nanomedicine, CAS Key Lab for Health Informatics, Shenzhen Engineering Laboratory of Nanomedicine and Nanoformulations, Shenzhen Institutes of Advanced Technology (SIAT), Chinese Academy of Sciences, Shenzhen, China; ^3^School of Materials Science and Engineering, Guilin University of Electronic Technology, Guilin, China; ^4^University of Chinese Academy of Sciences, Beijing, China; ^5^Faculty of Health Sciences, University of Macau, Macau, China; ^6^Dongguan Key Laboratory of Drug Design and Formulation Technology, Key Laboratory for Nanomedicine, Guangdong Medical University, Dongguan, China

**Keywords:** bimetallic nanoagents, photosensitizers, tumor microenvironment, reactive oxygen species, phototherapy

## Abstract

Phototherapy is a promising oncotherapy method. However, there are various factors greatly restricted phototherapy development, including poor tumor-specific accumulation, the hypoxia in solid tumor, and the systemic phototoxicity of photosensitizer. Herein, a tumor microenvironment (TME)-responsive intelligent bimetallic nanoagents (HSA-Pd-Fe-Ce6 NAs) composed of human serum albumin (HSA), palladium-iron (Pd-Fe) bimetallic particles, and chlorin e6 (Ce6) was designed for effective combination phototherapy. The Pd-Fe part in the HSA-Pd-Fe-Ce6 NAs would react with the endogenous hydrogen peroxide (H_2_O_2_) in an acidic ambiance within tumor to generate cytotoxic superoxide anion free radical through the “Fenton-like reaction.” H_2_O_2_, coupled with highly toxic singlet oxygen (^1^O_2_) caused by the Ce6 component under the irradiation of 660 nm laser, resulted in synergistic cancer therapy effects in hypoxia surroundings. Besides, this nanoagents could result in hyperpyrexia-induced cell apoptosis because of superior absorption performance in near-infrared wavelength window bringing about excellent photothermal conversion efficiency. The cell cytotoxicity results showed that the survival rate after treated by 40 μg mL^–1^ nanoagents was only 17%, which reveals that the HSA-Pd-Fe-Ce6 NAs had the advantage of efficient and controllable phototherapy. In short, it exhibited excellent hypoxia-resistant combination phototherapy efficacy *in vitro*. Therefore, the multifunctional nanoagents are powerful and provide a new avenue for effective combination phototherapy.

## Introduction

Cancer has become an important cause of morbidity and mortality worldwide. As the GLOBOCAN 2018 reported, there were 18.1 million new cases of cancer and 9.6 million deaths resulting from cancer in 2018 (Bray et al., 2018). The conventional cancer treatment options are chemotherapy, surgery resection, and radiotherapy. However, chemotherapy often comes with systemic side effects and high recurrence rate, which is associated with surgical resection (Sun et al., 2019). Meanwhile, radiation therapy is limited by the cumulative radiation dose. Therefore, it is an urgent issue to develop a smart, safe, efficient, and cost-effective alternative strategy to treat cancer (Liu et al., 2018; Pu et al., 2019).

As one of the non-invasive tumor therapy, phototherapy has become a hot research topic in recent years (Ma et al., 2020). It can be devided into two categories: photothermal therapy (PTT) and photodynamic therapy (PDT) (Meng et al., 2018; Zhou et al., 2018). PTT is an efficient cancer treatments that artificially elevates tissue temperatures to take advantage of cells with a weak defense against heat, causing minimal side effects. Many PTT delivery materials, including nanocomposites, have been developed for the applications in oncotherapy (Robinson et al., 2011; Jung et al., 2015; Lim et al., 2015; Hu et al., 2019). PDT, a typical phototherapy, involves the administration of a photosensitizer (PS) followed by local illumination of the tumor with light in a specific wavelength to active the PS, which converts molecular oxygen into cytotoxic reactive oxygen species (ROS) resulting in tumor cell ablation (Dougherty et al., 1975; Dolmans et al., 2003). Obviously, such a light-triggered and oxygen-dependent therapy has better selectivity to the target site and less toxic side effects on the biological system than the traditional therapy (Hu et al., 2016). However, neither PTT nor PDT can realize the perfect cancer treatment, since they have their own weakness. For example, most of the small molecule PSs such as phthalocyanines, porphin, and porphyrin derivatives which are currently available in the clinical practice (Brown et al., 2004) generally have various drawbacks, such as the lack of specificity, rapid metabolism, and phototoxicity (Hu et al., 2015). It is known that the rapid growth of tumor cells and abnormal vascularization would confine oxygen supply, aggravate hypoxia, and acidization tumor microenvironment (TME), which not only promote the angiogenesis and metastasis, but also could be main causes for the therapeutic resistance and failure of phototherapy (Semenza, 2003, 2012; Bertout et al., 2008; Rademakers et al., 2008; Lou et al., 2011) especially for PDT, which requires oxygen to be maximally cytotoxic. Therefore, it is highly demanded to develop intelligent phototherapy nanoagents that are sensitive to TME for better phototherapy effect.

**SCHEME 1:**
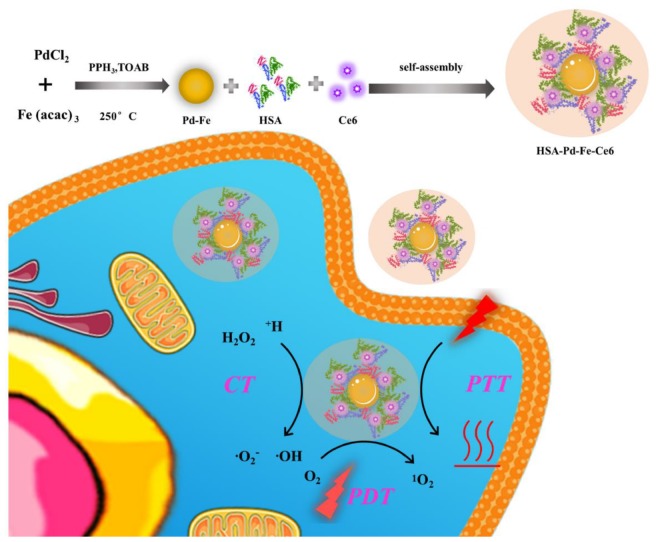
The process of HSA-Pd-Fe-Ce6 NAs synthesis and its application for synergistic phototherapy. The Pd-Fe NPs react with the endogenous hydrogen peroxide (H_2_O_2_) within tumor cell to generate cytotoxic superoxide anion free radical through the “Fenton-like reaction”. Moreover, Ce6 also turns O_2_ into a highly toxic singlet oxygen (^1^O_2_) by photodynamic reaction under 660 nm laser. Other than this, the nanoagents result in hyperpyrexia induced cell apoptosis.

In recent years, various strategies have been explored to overcome therapeutic resistance of phototherapy (Qing et al., 2019a), including the application of artificial blood substitutes (such as perfluorocarbons) to transport oxygen into tumors (Song et al., 2016) and inorganic nanocatalysts to generate oxygen *in situ* within the tumor (Prasad et al., 2014; Chen et al., 2015a). Moreover, Fenton-like catalytic reaction also plays an essential role in cancer therapy. For example, Liu et al. (2020) fabricated a biodegradable nanoscale coordination polymers for chemodynamic therapy. And Chen et al. (2019) developed AFeNPs@CAI nanocomposites to accelerate the Fenton reaction for amplified oxidative damage to cells. Herein, we have developed a self-assembling intelligent bimetallic nanoagents, HSA-Pd-Fe-Ce6 nanoagents (NAs) for effective combination phototherapy (Scheme 1). The HSA-Pd-Fe-Ce6 NAs are composed of human serum albumin (HSA), which is the most abundant plasma protein in human body and a multifunctional biocompatible drug delivery carrier to tumor (Xie et al., 2010; Elzoghby et al., 2012; Mertz et al., 2012a, b; Chen et al., 2014a, b, 2015b,c; Gause et al., 2015), palladium-iron bimetallic particles (Pd-Fe NPs) which have high reactivity toward hydrogen peroxide (H_2_O_2_) to genrate superoxide anion free radicals, and chlorin e6 (Ce6), a commercial photosensitizer, which converts molecular oxygen into cytotoxic singlet molecular oxygen (^1^O_2_) by PDT (Yoon et al., 2012; Huang et al., 2016; Liu et al., 2016). It is possible that the hydrophobic Ce6 and Fe-Pd nanoparticles enter the hydrophobic chamber of HSA, thereby forming an amphiphilic molecular system.

In this system, we took advantages of high loading capacity, biocompatibility of HSA, which could overcome the potential problems carused from Ce6, such as poor solubility in an aqueous solution and lack of tumor targeting. So this nanoagents were able to be enriched in tumor by passive targeting (enhanced permeability and retention effect). The experimental results demonstrated that subacid and excessive hydrogen peroxide of TME enabled Pd-Fe bimetallic NPs to generate hydroxyl radical by the “Fenton-like” reaction (Chand et al., 2009; Pang et al., 2011). Simultaneously, the Ce6 would convert oxygen into ^1^O_2_ under irradiation. In addition, the results indicated that HSA-Pd-Fe-Ce6 NAs could produce topical heat under the irradiation of 660 nm laser and near infrared absorption induced cell apotosis. Thus, this nanoagents could resolve the problems of traditional photosensitizer, such as poor solubility in aqueous solution, poor specific accumulation, and insufficient treatment effect. Therefore, the nanoagents are promising smart multifunctional integrated NAs for effective combination phototherapy.

## Materials and Methods

### Materials and Instruments

Ni(acac)_2_, PdCl_2_, oleylamine (OA, 90%), dichloromethane (DCM, 99%), Tetraoctylamine bromide (TOAB) were obtained from Sigma– Aldrich (United States). HSA was purchased from biomatrik (CHN). Triphenylphosphine (PPh_3_) and Tetrahydrofuran (THF) were obtained from Aladdin (CHN). Hoechst 33258 and Dihydroethidium (DHE) were purchased from Thermo Fisher Scientific (United States). Cell Counting Kit-8 (CCK-8) and 2,7-Dichlorofluorescein (DCFH-DA) were obtained from Dojindo laboratories (JPN). Penicillin–streptomycin, fetal bovine serum, PBS, DMEM medium, and trypsin were acquired from Gibco Life Technologies (United States). MCF-7 were obtained from Shanghai cell bank of Chinese Academy of Sciences.

#### Preparation of Pd-Fe

The Pd-Fe nanoparticles were synthesized by the previously reported procedure (Xiang et al., 2014; Chen et al., 2018). In short, a mixture of PdCl_2_ (0.01 g), Fe (acac)_3_ (0.04 g), Triphenylphosphine (50 mg) and TOAB (0.03 g) were dissolved in a mixed solution of tetrahydrofuran (5 mL) and oleylamine (6 mL), placed in a 100 mL three-neck round-bottom flask and was stirred at room temperature for 10 min. Then its temperature was increased to 90°C for 10 min. It was subsequently heated up to 250°C. After a 30 min reaction, the solution was cooled down to room temperature. Obtained Pd-Fe (Supplementary Figure S1) nanoparticles were washed and centrifuged several times with dichloromethane and then dispersed in the dichloromethane for further use.

#### Preparation and Characterization of HSA-Pd-Fe-Ce6

The as-prepared hydrophobic Pd-Fe nanoparticles were transferred into aqueous solution by coating with HSA amphiphilic copolymer. The Pd-Fe nanoparticles (10 mg mL^–1^, 500 μL) were mixed with powder of Ce6 (5 mg) and HSA (80 mg) in dichloromethane (2 mL) as solvent. Ultrapure water (2 mL) was added in to mixed solution forms a biphasic system. Then, nitrogen was bubbled in the solution and mixture changed cloudy to be an emulsion. After bubbled for 30 min, emulsion became clearly because of completely evaporation of the dichloromethane. As acquired HSA-Pd-Fe-Ce6 NAs were purified by a membrane filter (0.22 μm) to remove clustered large nanoparticles and a centrifugal filtration device (Millipore, *M*_*w*_ cutoff = 100 kDa) to get rid of excess HSA for several times. Purified HSA-Pd-Fe-Ce6 NAs were concentrated for further characterization and application. The UV–vis absorbance spectra and photoluminescence (PL) spectra of HSA-Pd-Fe-Ce6 NAs were measured using PerkinElmer Lambda 25 UV–vis absorption spectrophotometer and Edinburgh FS920 fluorescent spectrometer, respectively. TEM images of HSA-Pd-Fe-Ce6 NAs and Pd-Fe NPs were recorded using FEI Tecnai G20 transmission microscope at 200 kV. Dynamic light scattering (DLS) analysis was taken using a Zeta sizer Nano ZS (Malvern Instruments).

#### Photothermal Property of HSA-Pd-Fe-Ce6 Nanoagents

HSA-Pd-Fe-Ce6 (3.0 mg) nanoparticles were mixed with dichloromethane (2 mL) and irradiated (0.5 w/cm^2^, 660 nm, 2 min). Then the solution was cooled down to room temperature. And then repeat the above steps five times while using a thermal infrared hand-held viewer (Ti27, Fluke, United States) to record temperature changes every 10 s.

#### ROS Generation of HSA-Pd-Fe-Ce6 Nanoagents

HSA-Pd-Fe-Ce6 (3.0 mg) NAs and HSA-Ce6 NPs (3.0 mg) were mixed with DNA (1 mg/mL) and DHE (4 mg/mL) in PBS (2 mL, pH 5.5). The fluorescence intensity was measured using Edinburgh FS920 fluorescent spectrometer before and after mixing with H_2_O_2_ (1 mM) for 8 min, respectively. Group of HSA-Pd-Fe-Ce6 + H_2_O_2_: HSA-Pd-Fe-Ce6 (3.0 mg) NAs were mixed with DCFH-DA (4 mg/mL) and H_2_O_2_ (1 mM) in PBS (2 mL). Group of HSA-Pd-Fe-Ce6: HSA-Pd-Fe-Ce6 (3.0 mg) NAs were mixed with DCFH-DA (4 mg/mL) in PBS (2 mL). Group HSA-Ce6 + H_2_O_2_: HSA-Ce6 (3.0 mg) NPs were mixed with DCFH-DA (4 μg/mL) and H_2_O_2_ (1 mM) in PBS (2 mL). All groups were irradiated (660 nm, 0.5 w/cm^2^) for 30 s and then measured by Edinburgh FS920 fluorescent spectrometer per 30 s.

#### *In vitro* Cellular and 3D-Spheroids Cell Uptake of HSA-Pd-Fe-Ce6 Nanoagents

Flow cytometric assay and CLSM were employed to investigate the cell uptake. The typical procedure was described as follows. Tumor cell (MCF-7) were seeded in six-well plates and cultured for 24 h. The density of various cells is consistent before experiment. Then, the medium was replaced with fresh medium containing HSA-Pd-Fe-Ce6 NAs (1.6 μg mL^–1^) or HSA-Ce6 (1.6 μg mL^–1^). After 2 h incubation, cells were washed thrice with PBS and then digested by trypsin and harvested by centrifugation. The fluorescence of histograms of Ce6 were recorded by flow cytometer (Becton Dickinson, San Jose, CA, United States).

For CLSM observation (Qing et al., 2019b), MCF-7 cells (5000 cells per well) were seeded in eight-well chambered cover glasses (Lab-Tek, Nunc, United States). The old medium at 24 h was changed by the medium with HSA-Pd-Fe-Ce6 NAs (1.6 μg mL^–1^). After 2 h, PBS washed the cells thrice and then stained with Hoechst 33258 and PBS rinsed thrice. Finally, Leica TCS SP5 confocal laser scanning microscope (GER) was used to observe cellular uptake and subcellular distribution.

#### Intracellular ROS

The MCF-7 cells were seeded in six-well plate, incubated for 24 h in 5% CO_2_ at 37°C. Next, added to PBS, HSA-Ce6, HSA-Pd-Fe-Ce6 NAs and H_2_O_2_ (50 μM) in medium make the final concentrations consistent. After irradiation treatment (0.5 w/cm^2^ laser 20 min), cells were promptly washed with PBS and incubated with 10 μmol L^–1^ 2′,7′-dichloro-fluoreseindiacetate (DCFH-DA) and dihydroethidium for 30 min, and intracellular ROS generation was evaluated by flow cytometry.

#### Cytotoxicity Assay

For PTT, the MCF-7 cells were then incubated with HSA -Ce6 or HSA-Pd-Fe -Ce6 at a Ce6 equiv concentration from 0 to 40 μg mL^–1^ for 4 h. Then, treated with non-irradiated, irradiated (808 nm, 0.5 w/cm^2^, 20 min). In order to achieve PTT only effect, Vitamin C (2 × 10^–3^ M) was added to scavenge intracellular ROS.

For combined treatment (CBT), the MCF-7 cells were then incubated with HSA -Ce6 or HSA-Pd-Fe -Ce6 at a Ce6 equiv concentration from 0 to 40 μg mL^–1^ for 4 h. Then, treated with or without 50 μM H_2_O_2_ for 2 h.

For PDT, the MCF-7 cells were then incubated with HSA -Ce6 or HSA-Pd-Fe -Ce6 at a Ce6 equiv concentration from 0 to 40 μg mL^–1^ for 4 h. Then, treated with non-irradiated, irradiated (660 nm, 0.5 w/cm^2^, 20 min). In order to achieve PDT only effect, cells were cooled at 4°C during the irradiation.

For combination therapy, MCF-7 cells were incubated with HSA-Ce6 or HSA-Pd-Fe-Ce6 at a Ce6 equiv concentration from 0 to 40 μg mL^–1^ for 4 h. Then, treated with all above treatment.

After rinsing three times with PBS, all groups were incubated with new culture medium. CCK-8 assay was carried out to investigate the cell survival of different groups after 4 h. At 4 h post laser irradiation, 10 μL CCK-8 mixed with 90 μL of DMEM were added to each well of the plate. Incubated the plates for an hour in the incubate. Then measured the absorbance at 450 nm using a multimode microplate reader (Synergy4, BioTek, United States).

To obtain visible results, *in vitro* cell cytotoxicity of HSA-Ce6 and HSA-Pd-Fe-Ce6 NAs were also investigated by Dead cell staining via confocal laser scanning microscope (FV3000, Olympus, Japan) observation. MCF-7 cells (5 × 10^3^) were added into each well of a chamber slide with different treatment. Control: MCF-7 cells incubated with HSA -Ce6 or HSA-Pd-Fe -Ce6 for an hour. PTT: The cells incubated with HSA -Ce6 or HSA-Pd-Fe -Ce6 after irradiation (808 nm, 0.5 w/cm^2^) for an hour. In order to achieve PTT only effect, Vitamin C (2 × 10^–3^ M) was added to scavenge intracellular ROS. CT: The cells incubated with HSA -Ce6 or HSA-Pd-Fe-Ce6 after incubation with 50 μM H_2_O_2_ for 1 h. PDT: The cells incubated with HSA -Ce6 orHSA-Pd-Fe-Ce6 after irradiation (660 nm, 0.5 w/cm^2^) for an hour. In order to achieve PDT only effect, cells were cooled at 4°C during the irradiation. CBT: The cells incubated with HSA -Ce6 or HSA-Pd-Fe -Ce6 after above treatment. dividing into five groups [PBS, HSA-Ce6, HSA-Pd-Fe-Ce6, HSA-Ce6(+), and HSA-Pd-Fe-Ce6(+)] and cultured for 24 h. After that, the PBS, HSA-Ce6, and HSA-Pd-Fe-Ce6 NAs (20 μg mL^–1^) were added into relative wells of chamber slide. 2 h later, groups HSA-Ce6(+) and HSA-Pd-Fe-Ce6(+) were irradiated (660 nm, 0.5 W cm^–2^, 20 min). After incubation for another 2 h, the PI was added into the well. After 35 min, the cells were observed by a confocal laser scanning microscope.

#### Statistical Analysi*s*

All the results are reported as mean ± SD. The differences among groups were determined using one-way ANOVA analysis and student’s *t*-test; ^∗^*P* < 0.05, ^∗∗^*P* < 0.01, ^∗∗∗^*P* < 0.001.

## Results and Discussion

### Characteristics of HSA-Pd-Fe-Ce6 NAs

**FIGURE 1:**
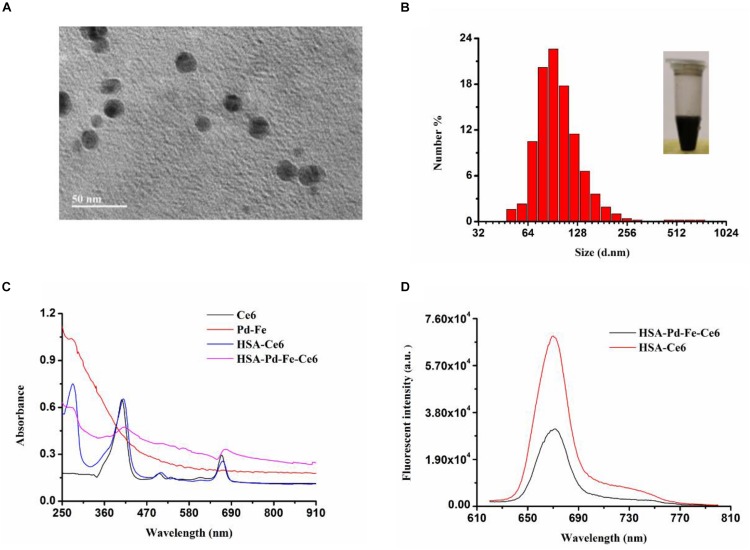
**(A)** A TEM image of HSA-Pd-Fe-Ce6. **(B)** Hydrodynamic diameters of HSA-Pd-Fe-Ce6 NAs and optical image (insert) of the nanoparticles. **(C)** Normalized UV–vis-NIR absorption spectra of Pd-Fe (dichloromethane), Ce6 (dichloromethane), HSA-Ce6 (Deionized water), and HSA-Pd-Fe-Ce6 (Deionized water) **(D)** fluorescence spectra at 600 nm of (a) HSA-Pd-Fe-Ce6 (70 μg/mL) and (b) HSA-Ce6 (70 μg/mL).

As revealed by transmission electron microscope (TEM) (Figure 1A), the synthesized HSA-Pd-Fe-Ce6 NAs showed average sizes at ≈20 nm and it can be observed that Pd-Fe NPs (deep black) are wrapped by HSA (light gray). As revealed by Malvern Particle Sizer, HSA-Pd-Fe-Ce6 NAs are stable and dispersed well in distilled deionized (DDI) water with an average NP size of 105 nm (Figure 1B). As illustrated by UV–vis spectra (Figure 1C), the Ce6 characteristic peaks (410 and 660 nm) and the characteristic peaks at 300 nm, which corresponded to HSA both are seen in the spectrum of the synthesized HSA-Pd-Fe-Ce6 NPs. It was also shown that HSA-Pd-Fe-Ce6 NAs have higher absorption than Ce6 and HSA-Ce6 absorption in the range of 700 and 1000 nm, similar to the way Pd-Fe NPs. The above mentioned results indicated the successful synthesis of HSA-Pd-Fe-Ce6 NAs in which Ce6 was conjugated onto Pd-Fe NPs coated with HSA. The absorption in the range of 750 to 1000 nm may render HSA-Pd-Fe-Ce6 NAs the photothermal effect that individual Ce6 does not have. At the same concentration, the fluorescence intensity stimulated by 600 nm of HSA-Ce6 NAs at 672 nm is more than twice that of HSA-Pd-Fe-Ce6 NAs (Figure 1D), which indicates that may have better phototherapy effect than HSA-Ce6 NAs. In terms of stability, the size of HSA-Pd-Fe-Ce6 NAs stabilized at 105 nm within a week (Supplementary Figure S2), while the size of HSA-Ce6 NAs maintained the trend of increasing (Supplementary Figures S3, S4).

**FIGURE 2:**
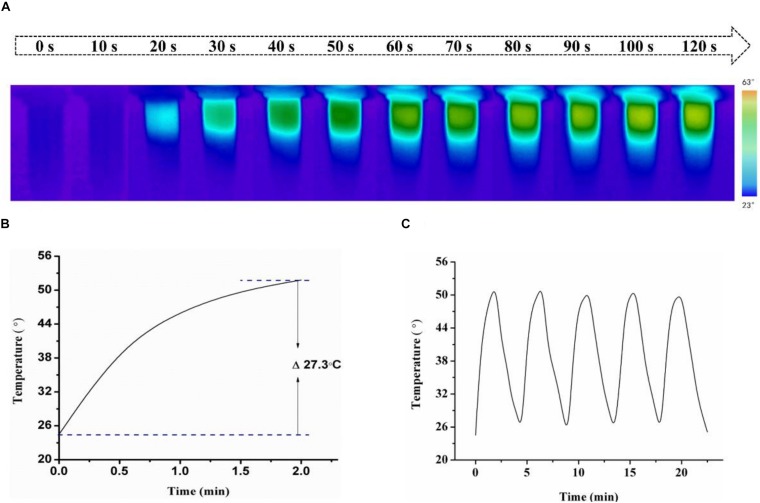
**(A)** Infrared images of HSA-Pd-Fe-Ce6 NAs (1.5 mg/mL) within 2 min under 808 nm laser (0.5 w/cm^2^) irradiation per 10 s and **(B)** Temperature growth curve of **(A)**. **(C)** A temperature cycling test of HSA-Pd-Fe-Ce6 NAs.

To investigate photothermal effect of HSA-Pd-Fe-Ce6 NPs, they were tested under the irradiation of 808 nm. As shown in Figures 2A,B, HSA-Pd-Fe-Ce6 NAs had increased in temperature up to 27.3°C and reached 51.8°C, which was high enough for tumor cell ablation. No obvious change was observed during the five cycles of heating/cooling, implying the great potential of HSA-Pd-Fe-Ce6 NAs as a durable photothermal agent (Figure 2C).

**FIGURE 3:**
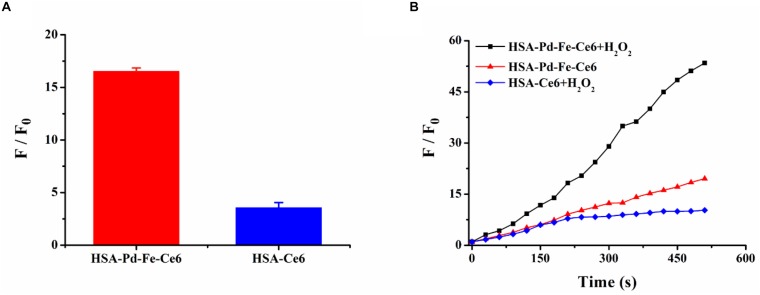
**(A)** The fluorescence intensity changes of dihydroethidium (DHE) between HSA-Pd-Fe-Ce6 NAs and HSA-Ce6 NPs after mixed with H_2_O_2_ (1 mM), DNA (1 mg/mL) and DHE (4 mg/mL) in PBS (pH 5.0). F_0_ refers to the fluorescence intensity before mixed with H_2_O_2_. **(B)** The fluorescence intensity changes of 2′,7′-dichlorofluorescein diacetate (DCFH-DA) between group HSA-Pd-Fe-Ce6 + H_2_O_2_, group HSA-Pd-Fe-Ce6 and group HSA-Ce6 + H_2_O_2_ NPs under 660 nm laser (0.5 w/cm^2^, 30 s) irradiation per 30 s. F_0_ refers to the fluorescence intensity before irradiation.

The ROS generation in solution was studied with superoxide anion (SOA) indicators, DHE and DCFH-DA. As shown in Supplementary Figure S5, though the group HSA-Ce6 exhibited a stronger fluorescence intensity after mixing with H_2_O_2_ compared with group HSA-Pd-Fe-Ce6, the latter showed more than four times relative intensity of the former implying great generation of SOD resulting from “Fenton-like” reaction (Figure 3A). To further investigate total ROS generation, group HSA-Pd-Fe-Ce6 + H_2_O_2_, HSA-Pd-Fe-Ce6 and HSA-Ce6 were studied with ROS indicator, DCFH-DA. As shown in Figure 3B and Supplementary Figure S6, group HSA-Pd-Fe-Ce6 + H_2_O_2_ showed nearly three times relative fluorescence intensity of group HSA-Pd-Fe-Ce6 and six times of HSA-Ce6 + H_2_O_2_ at 510 s point. In addition, intensity growth of group HSA-Ce6 seemed bog down since 270 s point while other two group seemed to continuous increase suggesting great and enduring ROS generation of HSA-Pd-Fe-Ce6.

### Uptake of HSA-Pd-Fe-Ce6 NAs

**FIGURE 4:**
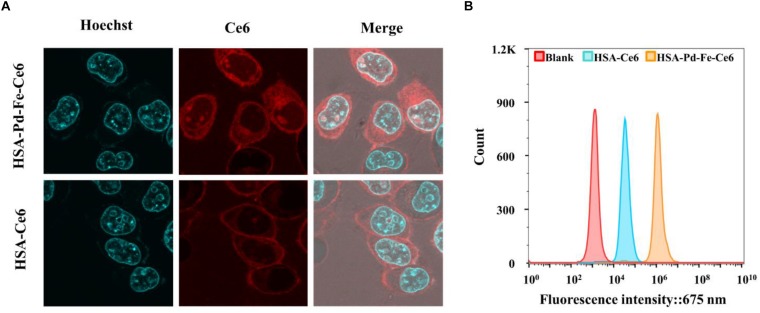
**(A)** The location of HSA-Pd-Fe-Ce6 in MCF-7. **(B)** Flow cytometric analysis of uptake of HSA-Pd-Fe-Ce6 and HSA-Ce6 by MCF-7 cells.

The human breast cancer cell line MCF-7 cells were incubated with HSA-Pd-Fe-Ce6 NAs or HSA-Ce6 NAs (20 μg mL^–1^) for 2 h at 37°C, and cell uptake was visualized using confocal laser scanning microscopy. The cell nucleus was stained greenish-blue to differentiate cellular compartments. The images show uniform distribution of HSA-Pd-Fe-Ce6 NAs and Ce6 in the cytoplasm and around the nucleus of the cells, while the former intracellular fluorescence intensity corresponding to assimilated particles amount was much higher than the latter (Figure 4A), indicating HSA enhanced the efficiency of the internalization of HSA-Pd-Fe-Ce6 NPs. To further certify it, flow cytometric analysis was carried out to analyze and quantify cellular uptake. The fluorescence intensity associated with Ce6 of HSA-Pd-Fe-Ce6 NAs was several orders of magnitude higher than Ce6 and blank (Figure 4B), which was consistent with the results above. These results demonstrate that HSA-Pd-Fe-Ce6 NAs was more easily taken up by tumor cells than free Ce6.

To understand the intratumoral transport behavior, *in vitro* 3D tumor spheroids were used to further investigate intratumoral transport of HSA-Pd-Fe-Ce6 NAs and HSA-Ce6. Tumor spheroids were formed using MCF-7 cells incubated with HSA-Pd-Fe-Ce6 NAs or HSA-Ce6 NAs. Confocal quantitative image cytometer CQ1 was used to image tumor spheroids every hour. As shown in Supplementary Figure S7, the fluorescence intensity of group HSA-Pd-Fe-Ce6 was obviously stronger than that from HSA-Ce6 at 4 h and the former maintained strong pink signal afterward while the latter was attenuated over time, indicating great intratumoral permeability and retention of HSA-Pd-Fe-Ce6 NAs.

### Intracellular Generation of ROS

It has been known that the vigorous metabolism and restricted blood supply for cancer cells resulting in significant increase of the H_2_O_2_ level in the tumor sites (Kuang et al., 2011), so a ROS probe 2′,7′-dichlorofluorescein diacetate (DCFH-DA) was then used to assessed the intracellular generation of ROS. MCF-7 cells were incubated with HSA-Ce6 NAs, HSA-Pd-Fe-Ce6 NAs, and H_2_O_2_ for 2 h, respectively, followed by incubation with DCFH-DA and treatment with the 660 nm laser. As shown in Supplementary Figure S8, The fluorescence intensity of HSA-Pd-Fe-Ce6 NAs was several orders of magnitude higher than HSA-Ce6 and blank, which was attributed to T the *in situ* production of ROS by the reaction of Pd-Fe with the H_2_O_2_ in the tumor under the acidic condition and the ROS produced by Ce6. Meanwhile, another superoxide indicator, dihydroethdium, was also used to evaluate the generation of superoxide anion free radical. It exhibits blue-fluorescence in the cytosol until oxidized in the nucleus, where it intercalates within the cell’s DNA and emits a bright red fluorescence. As shown in Supplementary Figure S9, MCF-7 cells, when incubated with 20 μg mL^–1^ HSA-Pd-Fe-Ce6 and H_2_O_2_, produced the strongest fluorescence signal, indicting the most efficient in the generation of intracellular superoxide anion free radical.

### Cytotoxicity Assay of HSA-Pd-Fe-Ce6 NAs

**FIGURE 5:**
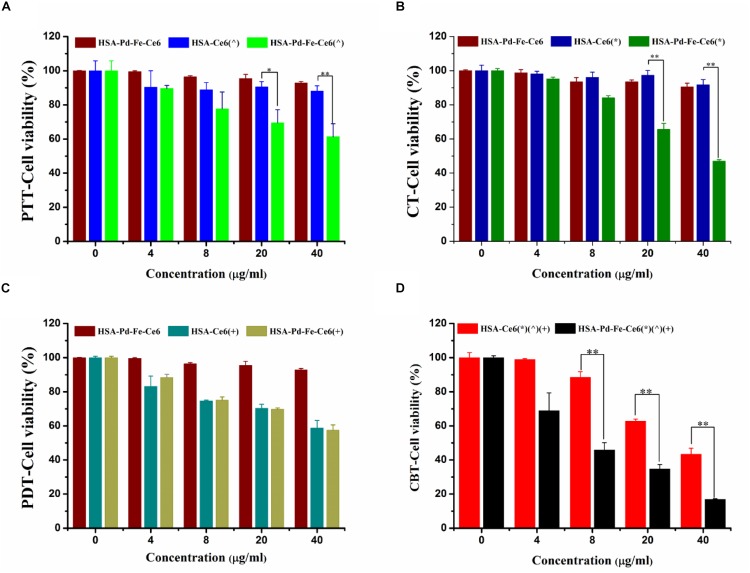
**(A)** The cell cytotoxicity of HSA-Pd-Fe-Ce6 only, HSA -Ce6 and HSA-Pd-Fe -Ce6 after irradiation (808 nm, 0.5 w/cm^2^) for an hour (*n* = 6). In order to achieve PTT only effect, Vitamin C (2 × 10^–3^ M) was added to scavenge intracellular ROS **(B)** The cell cytotoxicity of HSA-Pd-Fe-Ce6 only, HSA -Ce6 and HSA-Pd-Fe-Ce6 after incubation with 50 μM H_2_O_2_ for 4 h (*n* = 6). **(C)** The cell cytotoxicity of HSA-Pd-Fe-Ce6 only, HSA-Ce6 and HSA-Pd-Fe-Ce6 after irradiation (660 nm, 0.5 w/cm^2^) for an hour (*n* = 6). In order to achieve PDT only effect, cells were cooled at 4°C during the irradiation **(D)** The cell cytotoxicity of MCF-7 incubated with HSA -Ce6 or HSA-Pd-Fe -Ce6 after above treatment (*n* = 6). (^∧^) refers to 808 nm laser irradiation. (^+^) refers to 660 nm laser irradiation. (^∗^) refers to incubation with 50 μM H_2_O_2_ for 4 h. *P*-values were calculated by Tukey’s post-test (^∗∗^*P* < 0.01 or ^∗^*P* < 0.05). The error bars represent the standard error of six independent measurements.

*In vitro* cell cytotoxicity of HSA-Pd-Fe-Ce6 NAs was investigated using Cell Counting Kit-8 (CCK-8). The amount of water-soluble formazan was directly proportional to the number of living cells. To investigate the PTT effect of HSA-Pd-Fe-Ce6 NAs, MCF-7 cells were incubated with HSA -Ce6 or HSA-Pd-Fe -Ce6 at a Ce6 equiv concentration from 0 to 40 μg mL^–1^ for 4 h. Then, treated with non-irradiated, irradiated (808 nm, 0.5 w/cm^2^, 20 min). In order to achieve PTT only effect, Vitamin C (2 × 10^–3^ M) was added to scavenge intracellular ROS. Compared with other groups, group HSA-Pd-Fe-Ce6 under irradiation had significantly lower cell viability with about 61% cells survived when the concentration reached 40 μg mL^–1^, as shown in Figure 5A. For CBT, the MCF-7 cells were incubated with HSA -Ce6 or HSA-Pd-Fe -Ce6 at a Ce6 equiv concentration from 0 to 40 μg mL^–1^ for 4 h. Then, treated with or without 50 μM H_2_O_2_ for 2 h. Group HSA-Pd-Fe-Ce6 with H_2_O_2_ showed significantly low cell viability up to around 47% at 40 μg mL^–1^ concentration while the other groups kept considerable high percent survival within investigated concentration seen in Figure 5B. We could see the negative results of group HSA-Pd-Fe-Ce6 without treatment and HSA-Ce6 with relative treatment in both studies comparing with positive results of group HSA-Pd-Fe-Ce6 with treatment, implying Pd-Fe bimetallic part with irradition and Pd-Fe with H_2_O_2_ were the sufficient condition of PTT and CBT, respectively. To investigate PDT effect, MCF-7 cells were incubated with HSA -Ce6 or HSA-Pd-Fe -Ce6 at a Ce6 equiv concentration from 0 to 40 μg mL^–1^ for 4 h. Then, treated with non-irradiated, irradiated (660 nm, 0.5 w/cm^2^, 20 min). In order to achieve PDT only effect, cells were cooled at 4°C during the irradiation. As seen in Figure 5C, group HSA-Pd-Fe-Ce6 and HSA-Ce6 with irradiation showed identical results, which was because of the equivalent Ce6. For the combination therapy, MCF-7 cells were incubated with HSA-Ce6 or HSA-Pd-Fe-Ce6 at a Ce6 equiv concentration from 0 to 40 μg mL^–1^ for 4 h. Then, treated with all above treatment. As shown in Figure 5D, both groups showed positive outcomes but group HSA-Pd-Fe-Ce6 possessed significant lower cell survival than group HSA-Ce6 had from 8 to 40 μg mL^–1^ concentration and at the 40 μg mL^–1^ concentration the survival rate of groups were 17 and 43%, which were consistent to results above, revealing that the HSA-Pd-Fe-Ce6 NAs had the advantage of efficient and controllable phototherapy.

**FIGURE 6:**
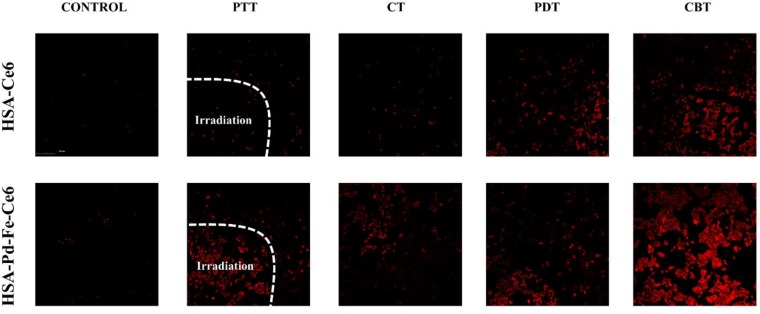
The Dead cell staining results of HSA-Ce6 NPs, HSA-Pd-Fe-Ce6 NPs with different treatment. Control: MCF-7 cells incubated with HSA -Ce6 or HSA-Pd-Fe -Ce6 for an hour. PTT: The cells incubated with HSA -Ce6 or HSA-Pd-Fe -Ce6 after irradiation (808 nm, 0.5 w/cm^2^) for an hour. In order to achieve PTT only effect, Vitamin C (2 × 10^– 3^ M) was added to scavenge intracellular ROS. CT: The cells incubated with HSA -Ce6 or HSA-Pd-Fe-Ce6 after incubation with 50 μM H_2_O_2_ for 1 h. PDT: The cells incubated with HSA -Ce6 orHSA-Pd-Fe-Ce6 after irradiation (660 nm, 0.5 w/cm^2^) for an hour. In order to achieve PDT only effect, cells were cooled at 4°C during the irradiation. CBT: The cells incubated with HSA -Ce6 or HSA-Pd-Fe -Ce6 after above treatment.

We further investigated *in vitro* cell cytotoxicity of HSA-Pd-Fe-Ce6 and HSA-Ce6 by dead cell staining, in which red fluorescent PI were used as dyes. As shown in Figure 6, groups HSA-Pd-Fe-Ce6 with treatment showed obvious red signal, implying cellular death were positive. Especially CBT treatment, a huge area was stained. However, there was barely red single seen in the group HSA-Ce6 with PTT or CT treatment. Though the area of group HSA-Ce6 with PDT treatment was the same as the area of HSA-Pd-Fe-Ce6, the area of HSA-Ce6 with CBT treatment was significantly smaller than HSA-Pd-Fe-Ce6’s, which were in agreement with the CCK8 assay.

In order to test the potential application of HSA-Pd-Fe-Ce6 NPs, they were injected subcutaneously into left lower abdomen of the mouse imaging by micro-ultrasound Imaging System. As shown in Supplementary Figure S10, HSA-Pd-Fe-Ce6 NPs had near-infrared (NIR) absorption photoacoustic signals from 700 to 970 nm. Moreover, it was clear that the longer the wavelength was, the less the background signals were, and the signal intensity didn’t weaken much at NIR wavelength. Unlike the visible spectrum, in which most tissue chromophores (hemoglobin, melanin, fat etc.) absorb light strongly, wavelength of the irradiated beam in the NIR region has a deeper tissue penetration into tissues. HSA-Pd-Fe-Ce6 is a promising photoacoustic contrast agent, which makes it possible to realize further applications, such as real-time imaging guidance and therapeutic evaluation.

## Conclusion

In conclusion, we have constructed self-assembling intelligent bimetallic nanoagents for effective combination phototherapy via reductive co-precipitation method. The presence of Pd-Fe and Ce6 offers HSA-Pd-Fe-Ce6 NAs two ways to generate ROS leading to superior ROS production, in which superoxide anion free radicals are produced by the reaction between Pd-Fe bimetallic NPs and H_2_O_2_ in acidic TME and singlet molecular oxygen converted from molecular oxygen though photodynamic reaction caused by the Ce6. Additionally, the results show that HSA-Pd-Fe-Ce6 NAs can produce topical heat under the irradiation and near infrared absorption bringing about excellent photothermal conversion efficiency. Overall, this nanoagents could resolve the problems of traditional photosensitizer, such as poor solubility in aqueous solution, poor specific enrichment, and insufficient treatment effect. Therefore, the nanoagents are promising smart multifunctional integrated nanophotosensitive agent for effective combination phototherapy.

## Data Availability Statement

All datasets generated for this study are included in the article/Supplementary Material.

## Ethics Statement

The animal study was reviewed and approved by Chinese Academy of Sciences.

## Author Contributions

HL and YL: data curation, validation, visualization, investigation, writing-original draft. JX, XY, CnL, CaL, QZ, LZ: writing-original draft. PG: funding acquisition, project administration, methodology, writing-review and editing. JH: funding acquisition, project administration, supervision, writing-review and editing.

## Conflict of Interest

The authors declare that the research was conducted in the absence of any commercial or financial relationships that could be construed as a potential conflict of interest.

## Supplementary Material

FIGURE S1 A TEM image of Pd-Fe.

FIGURE S2 Stability of HSA-Pd-Fe-Ce6 NAs in DI water.

FIGURE S3 Stability of HSA-Ce6 NAs in DI water.

FIGURE S4 Size change curve of S2 and S3.

FIGURE S5 The fluorescence intensity of dihydroethidium (DHE) of **(A)** HSA-Ce6 NPs and **(B)** HSA-Pd-Fe-Ce6 NAs after mixed with H_2_O_2_ (100 μM), DNA (1 mg/mL) and DHE (4 mg/mL) in PBS (pH 5.5). 0: refers to the fluorescence intensity before mixed with DHE.

FIGURE S6 The fluorescence intensity of 2′,7′-dichlorofluorescein diacetate (DCFH-DA) **(A)** group HSA-Pd-Fe-Ce6 + H_2_O_2_, **(B)** group HSA-Pd-Fe-Ce6, and **(C)** group HSA-Ce6 + H_2_O_2_ NPs under 660 nm laser (0.5 w/cm^2^, 30 s) irradiation per 30 s.

FIGURE S7 Images of the 3D spheroids of MCF-7 cells incubated with HSA-Pd-Fe-Ce6 or HSA-Ce6 per hour.

FIGURE S8 The generation of ROS determined by the DCFH-DA fluorescence of MCF-7 cells after coincubated with PBS or HSA-Ce6 nanoparticles (20 μg/mL) or HSA-Pd-Fe-Ce6 NAs (20 μg/mL) with H_2_O_2_ (50 μM) for 2 h and further 20 min laser irradiation.(660 nm,0.5 w/cm^2^).

FIGURE S9 Use of a free radical indicator to evaluate the production of ROS (⋅OH,⋅O_2_^–^) after incubation of MCF-7 cells with dihydroethidium and H_2_O_2_ (50 μM).

FIGURE S10 Photoacoustic signals of HSA-Pd-Fe-Ce6 nanoparticles at 700, 760, 820, 880, 930, and 970 nm.
