# Supplementary material for: Intelligent Bimetallic Nanoagents as Reactive Oxygen Species Initiator System for Effective Combination Phototherapy
Source: Front Bioeng Biotechnol. 2020 May 8;8:423. doi: 10.3389/fbioe.2020.00423 (PMC7225307; doi:10.3389/fbioe.2020.00423)
Supplement: FIGURE S1 — A TEM image of Pd-Fe. [file Image_1.pdf]

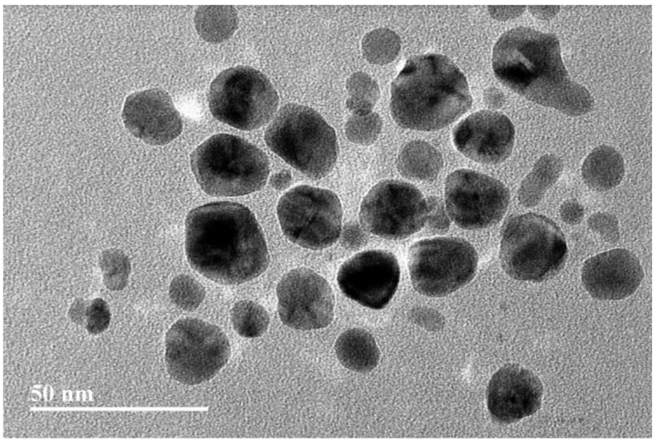

Fig. S1 A TEM image of Pd-Fe.

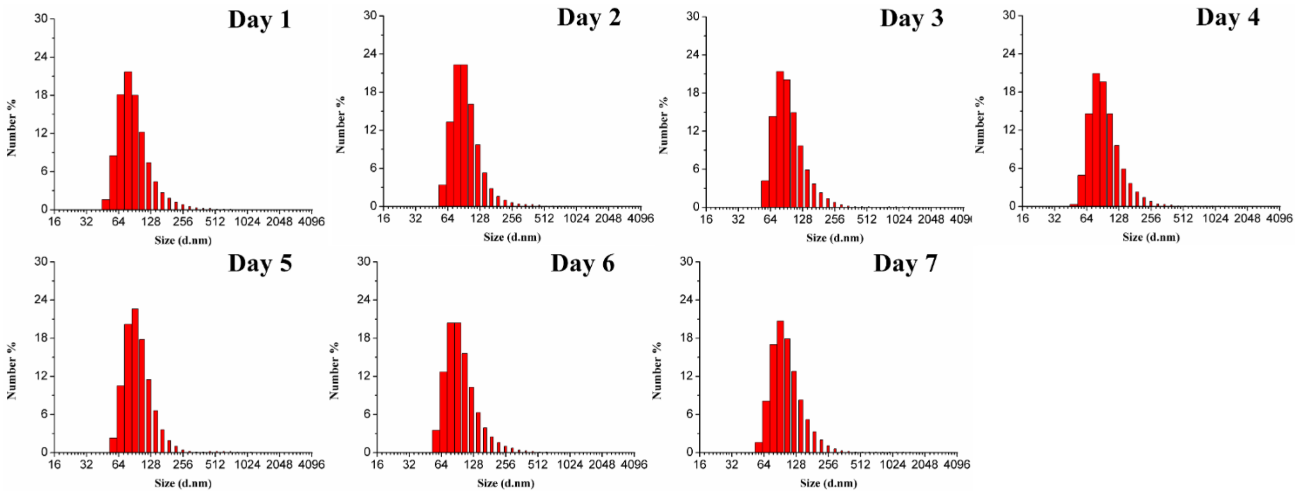

Fig. S2 Stability of HSA-Pd-Fe-Ce6 NAs in DI water**.**

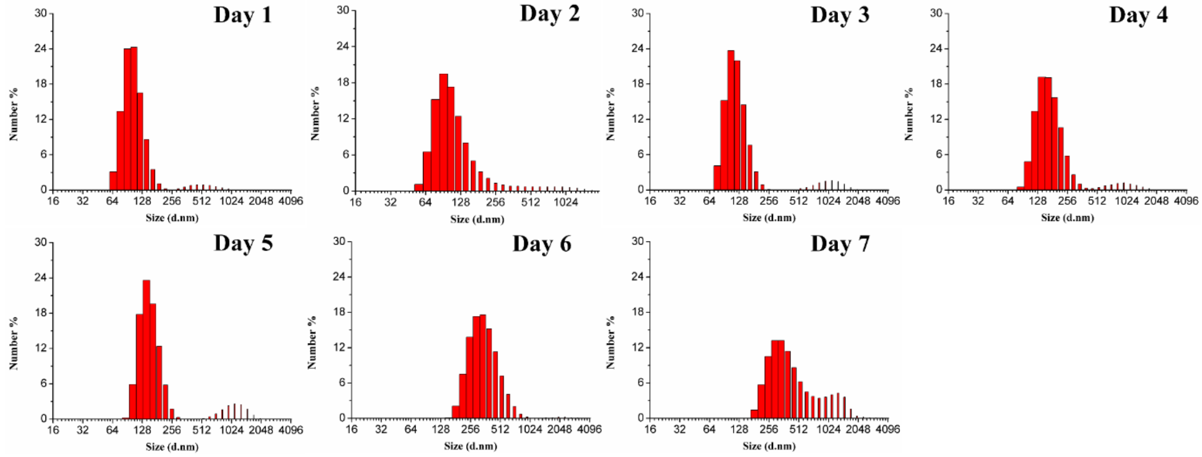

Fig. S3 Stability of HSA-Ce6 NAs in DI water.

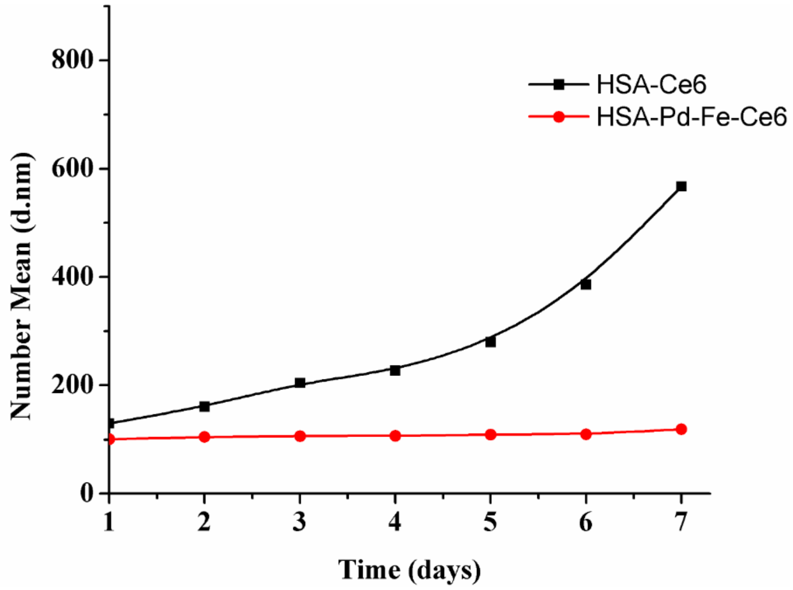

Fig. S4 size change curve of **S2** and **S3**.

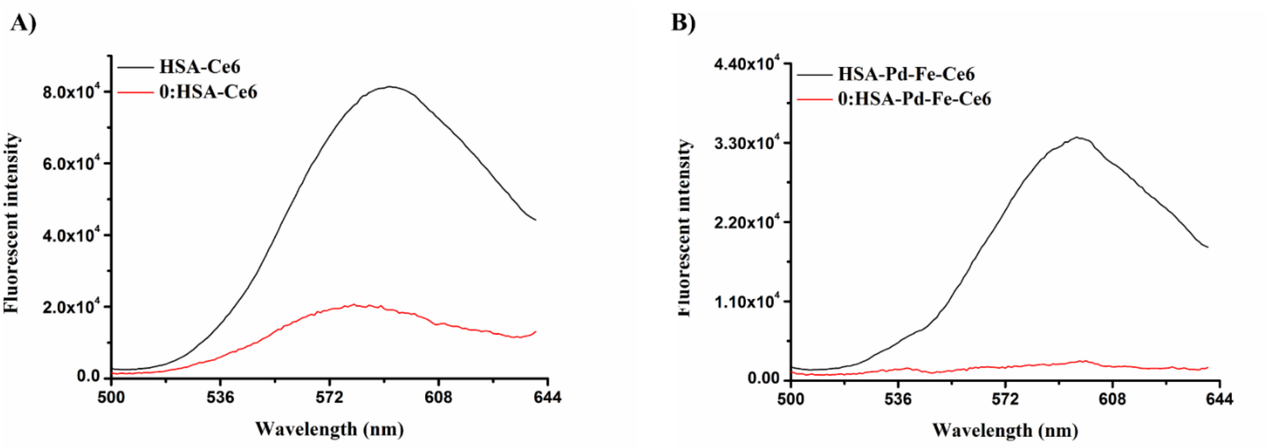

Fig. S5 The fluorescence intensity of dihydroethidium (DHE) of **A)** HSA-Ce6 NPs and **B)** HSA-Pd-Fe-Ce6 NAs after mixed with H_2O_2 (100 μM), DNA (1 mg/ml) and DHE (4 mg/ml) in PBS (pH 5.5). 0: refers to the fluorescence intensity before mixed with DHE.

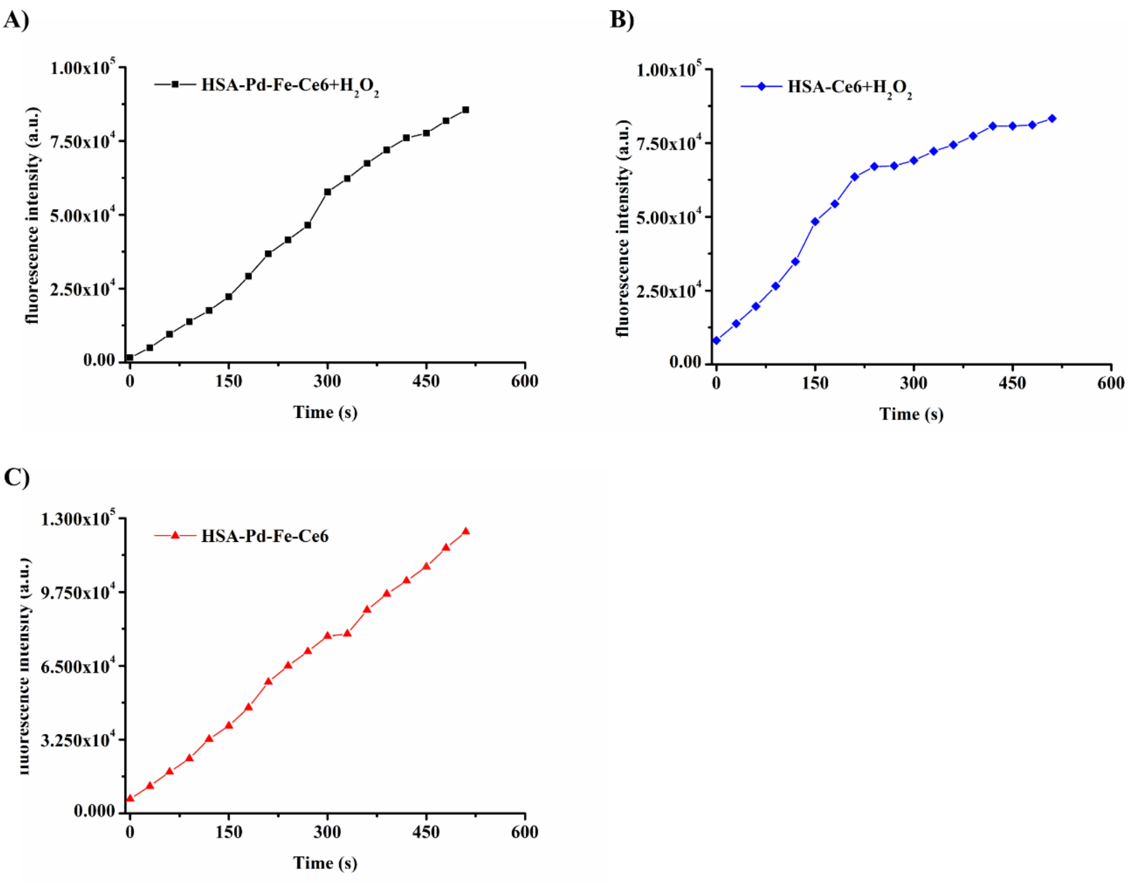

Fig. S6 The fluorescence intensity of 2′ ,7′ -dichlorofluorescein diacetate (DCFH-DA) **A)** group HSA-Pd-Fe-Ce6 +H_2O_2, **B)** group HSA-Pd-Fe-Ce6 and **C)** group HSA-Ce6+H_2O_2 NPs under 660 nm laser (0.5 w/cm^2, 30 s) irradiation per 30 s.

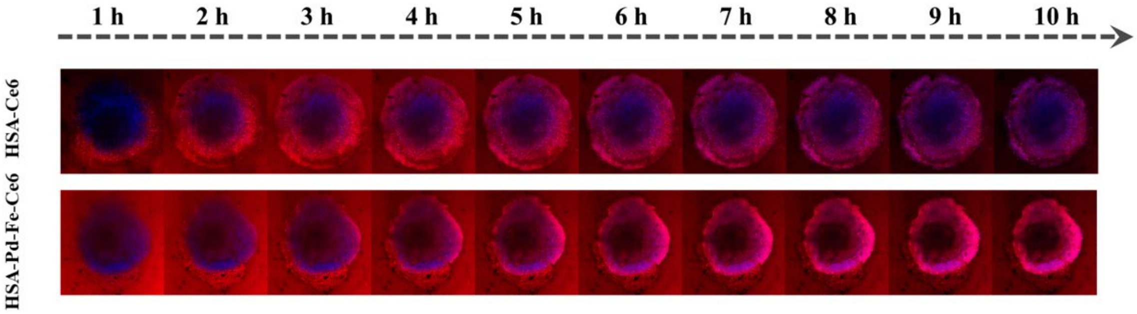

Fig. S7 Images of the 3D spheroids of MCF-7 cells incubated with HSA-Pd-Fe-Ce6 or HSA-Ce6 per hour.

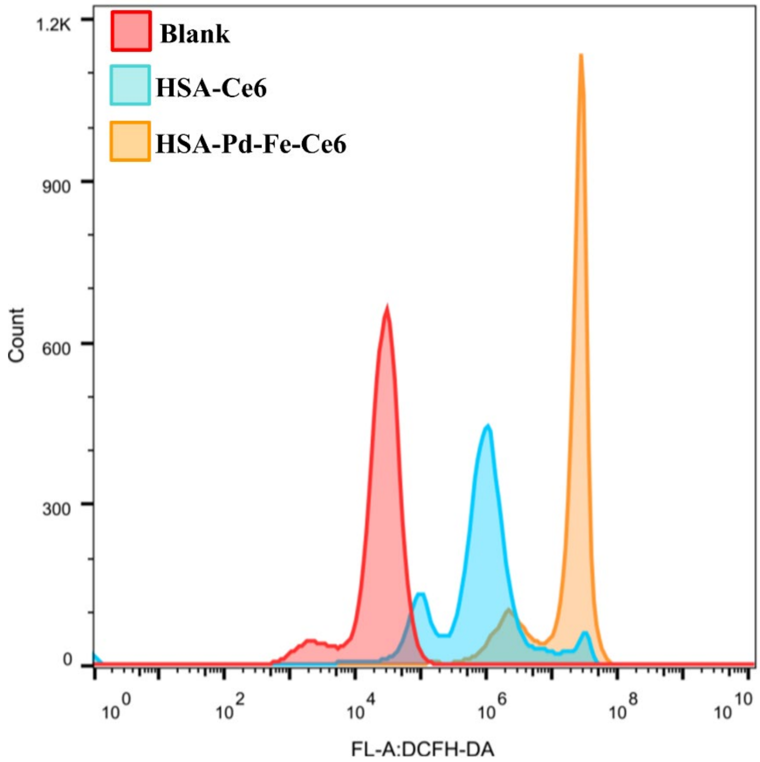

Fig. S8 The generation of ROS determined by the DCFH-DA fluorescence of MCF-7 cells after coincubated with PBS or HSA-Ce6 nanoparticles (20 μg/ml) or HSA-Pd-Fe-Ce6 NAs (20 μg/ml) with H_2O_2 (50 μM) for 2 h and further 20 minutes laser irradiation.(660 nm,0.5 w/cm^2).

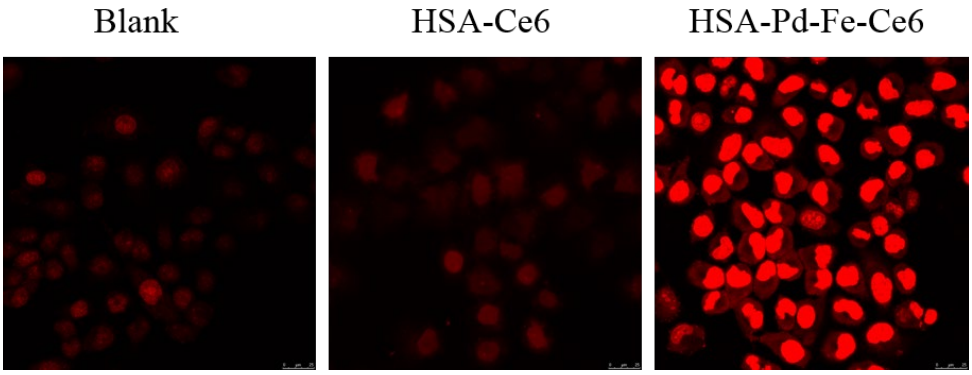

Fig. S9 Use of a free radical indicator to evaluate the production of ROS (·OH, ·O_2^-) after incubation of MCF-7 cells with dihydroethidium and H_2O_2 (50 μM).

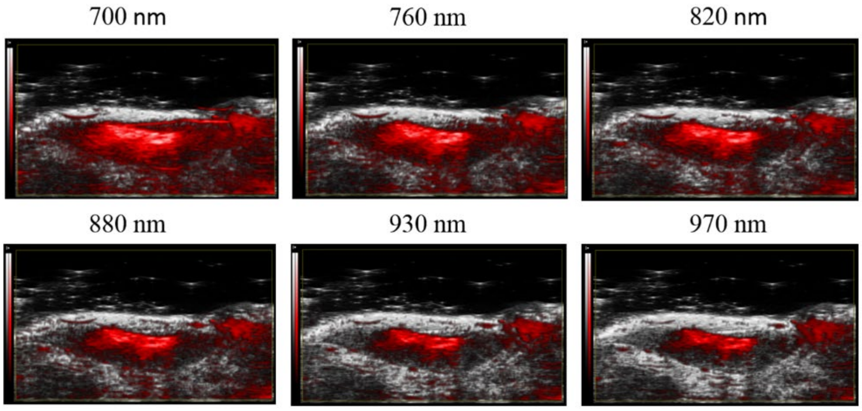

Fig. S10 Photoacoustic signals of HSA-Pd-Fe-Ce6 nanoparticles at 700 nm, 760 nm, 820 nm, 880 nm, 930 nm and 970 nm.
